# Medical Jurisprudence

**Published:** 1837-04

**Authors:** 


### MEDICAL JURISPRUDENCE.

### *On the Morbid Appearances in Death by Drowning.* By W. Ogston, m.d. Aberdeen.

Dr. Ogston has endeavoured to estimate, by the numerical method, the characteristic signs and morbid appearances of seventeen cases of death by drowning, which came under his notice, during the last six years, in police practice. The bodies were examined in seven cases. The following are the principal results and remarks:

1. The only phenomena which were all but universal, were dilated pupils, clenched jaws, and semi-contracted fingers and thumbs. 2. There was commonly at first paleness of the face, which, after a longer or shorter interval, became livid. 3. Striking calmness and placidity of the features. 4. Excoriation at the ends of the fingers, and dirt or sand under the nails, which have been laid down as frequent marks of death by drowning, were not met with. 5. The tip of the tongue was usually found in accurate contact with the incisor teeth; in two instances included between the closed jaws, but not wounded. 6. In seven cases only was there froth about the mouth; an appearance on which much stress has been laid. 7. Froth was met with in the mouth in two cases only of those dissected; and in three only in the trachea. 8. Liquidity of the blood has been remarked; in five of the bodies examined, coagulated blood was found in the heart, though the great mass of blood was fluid. 9. Water was found in the stomach in almost all the cases examined.

M. Devergie has lately endeavoured to show that, by certain appearances, the time may be determined which has elapsed since submersion: as might be expected, Dr. Ogston's cases do not confirm M. Devergie's statements.

*Edinburgh Med. and Surg. Journal, Jan.* 1837.

